# Prediction of Lipophilicity and Pharmacokinetics of Chloroacetamides by Chemometric Approach

**Published:** 2018

**Authors:** Gyöngyi Vastag, Suzana Apostolov, Borko Matijević

**Affiliations:** *Department of Chemistry, Biochemistry and Environmental Protection, Faculty of Sciences, University of Novi Sad, Trg Dositeja Obradovića 3, 21000 Novi Sad, Serbia.*

**Keywords:** *N*-(substitutedphenyl)-2-chloroacetamides, Lipophilicity, RPTLC, Pharmacokinetic predictors, Cluster Analysis, Principal Component Analysis

## Abstract

In this study, the existence of biological potential of selected *N*-(substituted phenyl)-2-chloroacetamides was examined none empirically, as was the possibility of applying simple experimental technique in predicting essential properties which affect the biological activity of the compounds. By applying the Lipinski and Ghose’s rules, it has been revealed that the examined chloroacetamides fulfill the theoretical requirements for bioactive compounds. In addition, lipophilicity was determined by applying the reversed-phase thin-layer chromatography (RPTLC_18F254s_) in the mixtures of water and two organic modifiers separately (methanol and acetone) and by using relevant software packages. The chromatographic retention parameters, *R*_M_^0 ^and *m*, as the presumed criteria for the lipophilicity of the examined chloroacetamides were correlated by linear regression analysis, and the relevant chemometric methods (Cluster Analysis and Principal Component Analysis) with the standard measure of lipophilicity, log *P*, and with the selected pharmacokinetic predictors. Thus good correlations in both water-modifier systems (average correlation coefficients, r , 0.947 and 0.931) were obtained. The chemometric methods, as well as the classical correlation methods gave similar results which demonstrated that the chromatographic retention parameters, *R*_M_^0 ^and *m*, can successfully describe the lipophilicity and the pharmacokinetics of the *N*-(substituted phenyl)-2-chloroacetamides in the first steps of preclinical research.

## Introduction

The task of contemporary research is the synthesis of compounds that may exhibit some biological activity. It has been found that most of the bioactive compounds synthesized during the last decade belong to the class of amides (1-5). Among numerous structurally different amide derivatives, the acetamides evince the most diverse pharmacological potential. Their characteristics make them applicable in numerous fields of herbal, veterinary, and human medicine. They have proved as fungicides (6), insecticides (7), bactericides (8), analgesics (9), anticonvulsants (10), antioxidants (11), antituberculotics (12), antidepressants (13), and antitumor agents (14-16). It has also been confirmed that some of acetamides are responsible for the regulation of body temperature (17) and apetite (18). The indisputable fact is that the type and intensity of activity of the acetamides is conditioned by the nature of the substituent attached to the basic molecule, as well as by the kind of interactions that the examined compound achieved at the site of action with the environment (19). Discovering, examining, and developing bioactive candidates (drug) with adequate properties carries a lot of risk, investment, and time (20). Bearing this in mind, the rationalization of future agent design could be accomplished by establishing dependencies between its structure, physicochemical properties and activity. The recognition and selection of relevant molecular descriptors which could be linked to the biological activity make this easier. For that purpose the often applied drug-likeness index are the Lipinski’s rule of five (Ro5) and the Ghose’s rule (GR) (21-25). Among numerous molecular descriptors, lipophilicity acts as the key one for the estimation of the biological activity of the compound. It determines the passage of compounds through the biomembranes and it is usually defined by the partition coefficient, log *P* (logarithm of the ratio of the concentrations of solute in the system 1-octanol-water) (26, 27). Apart from the standard criteria of lipophilicity, log *P*, chromatographic retention parameters, *R*_M_^0^ and *m*, obtained by RPTLC as alternative measures of lipophilicity have been applied (28-32). Also, lipophilicity is closely related to the pharmacokinetic properties (absorption, distribution, metabolism and elimination-ADME) of compound and thereby defines its bioavailability and effectiveness in the organism (33, 34). 

Taking into account the diversity of the biological activity of acetamides, the objects of this study are derivatives of *N*-(substituted phenyl)-2-chloroacetamides. Firstly, the studied compounds were checked whether they fulfill the conditions of drug-likeness within the rules of Lipinski and Ghose. Also, the influence of the molecule’s structure and the applied organic modifier on the lipopohilicity determined by RPTLC was examined. In order to investigate the possibility of application of the chromatographic parameters of the tested acetamides as measures of their lipophilicity and bioavailability, *R*_M_^0 ^and *m* were correlated with partition coefficient, log *P*, as well as with important pharmacokinetic parameters (*P*_eff_, *PPB* and log *BBB*) by linear regression analysis. A more detailed insight into the (dis)similarities between parameters of the Lipinski/Ghose, chromatographically/mathematically determined lipophilicity and pharmacokinetic properties of *N*-(substituted phenyl)-2-chloroacetamides was acquired by applying two methods of multivariate analysis: cluster analysis, CA, and principal component analysis, PCA.

## Experimental

*Materials and Methods*

The structures of the examined derivatives are presented in Table 1. During chromatographic examinations commercial plates (RP-18W/UV_254_ Macherey-Nagel GmBH and Co., Duren, Germany) were used as the stationary phase. Mixtures of LC grade organic modifiers (J. T. Baker, Deventer, the Netherlands) and filtered bi-distilled water were used as the mobile phase.

*RPTLC conditions*

The tested chloroacetamides were dissolved in ethanol (analytical purity) in concentration 2 mg mL^–1^. About 0.2 µL of freshly prepared solution of each compound was spotted on the plates which were then developed by the ascending technique in the unsaturated chambers at room temperature with an aqueous solutions of two organic modifiers: methanol (φ = 0.34 - 0.56, v/v) and acetone (φ = 0.34 - 0.56, v/v). The visualization of the tested compounds on the dried developed plates was performed under a UV light at λ= 254 nm. In both applied modifiers for each composition three chromatograms were developed and then the average *R*_f_ values were calculated.

*Processing of results*

The obtained experimental data were processed by Origin 6.1 software. The values of the partition coefficient, log *P*, were calculated using virtual Computational Chemistry Laboratory, VCCLAB (35). Molecular descriptors and values of pharmacokinetic predictors were obtained using Molinspiration, ChemSilico and SimulationPlus online programs (36-38). The CA and PCA procedures were performed by Statistica v.12 software (StatSoft Inc., Tulsa, OK, USA). 

## Results and Discussion

*Accordance of N-(substituted phenyl)-2-chloroacetamides with the drug-likeness rules*

Relying on the already confirmed biological activity of many chloroacetamide derivatives, the possibility that the studied *N*-(substituted phenyl)-2-chloroacetamides have bioactive potential was examined unempirically by two drug-likeness index: the Lipinski’s rule of five and the Ghose’s rule. According to the Lipinski’s rule of five, a potentially biologically active substance should have: molecular weight ≤ 500; number of hydrogen bond donor ≤ 5; number of hydrogen bond acceptor ≤ 10 (2×5) and values of calculated log *P *≤ 5. Similarly, in compliance with the Ghose’s rule a bioactive agent should possess: molecular weight within 160-480; values of calculated log *P *between -0.4 and 5.6; the total number of atoms in molecule within 20-70 and molar refractivity in the range 40-130 (21, 22). 

In Tables 2 and 3 the appropriate molecular descriptors of the investigated chloroacetamides are listed.

It is noticeable from Table 3 that for the same compound different values of partition coefficient, log *P*, as standard measure of lipophilicity, were obtained. This is the result of applying different mathematical methods for calculating log *P*. Independently of the mathematical calculating process the highest value of partition coefficient was obtained for compound with -I as substituent, and the lowest value was registered for compound with -OH group. Also, almost no difference was found for compound with the same substituent in different positions in the values of partition coefficients (-Br and -CN).

In total, the data shown in Tables 2 and 3 indicate that the examined derivatives have theoretical predisposition for some activity in biological medium because they fulfill the Lipinski’s rule of five and also mainly fit into the Ghose’s rule. Due to the fact that lipophilicity represents the most common indicator of the biological activity of the compound, it was further examined in more detail for derivatives of chloroacetamides.

*Determination of lipophilicity of chloroacetamide derivatives by RPTLC*

The interactions which the compound makes with its surroundings are very similar to those that it can establish with mobile and stationary phase during chromatographic analysis. With this in mind, evaluating lipophilicity of the tested chloroacetamides was carried out by RPTLC in water mixtures of one protic solvent (methanol) and one aprotic solvent (acetone) separately, with changing amount of the used solvents. 

From the experimentally obtained *R*_f_ values, for each composition of the mobile phase, *R*_M_ values were calculated according to the Equation:

 (1) $$R_{M}=\log\left(\frac{1}{R_{f}}-1\right)$$

The relationships between the retention values, *R*_M_, of the compound and the volume fraction of the organic modifier in the mobile phase, *φ*, were given by:

 (2) $$R_{M}=R_{M}^{0}+\mathrm{m\varphi}$$

Where intercept, *R*_M_^0^, represents the chromatographic retention constant, while the slope of the linear plot, *m*, which is the change in *R*_M _is caused by the unit of the organic modifier concentration in the mobile phase and depends on the properties of the solute. Commonly, it is related to the specific hydrophobic surface area of the compound (39). Chromatographic parameter *m* can be applied as alternative measure of lipophilicity, also (40, 41).Values of these parameters obtained for chloroacetamides in the used modifiers are given in Table 4.

High values of the regression coefficients, r, confirmed the validity of the linear dependencies in the chosen field of experimental work, were established between the retention parameter, *R*_M_, and the volume fraction of the organic modifier, *φ*.

It can be noticed from Table 4 that the nature of the substituent affects more the values of chromatographic parameters *R*_M_^0^ and *m* of analyzed chloroacetamides than the used organic modifier. Moreover, *m* values follow values of *R*_М_^0 ^and therefore the existence of the relations between these chromatographic parameters was assumed (Table 5).

**Figure 1:**
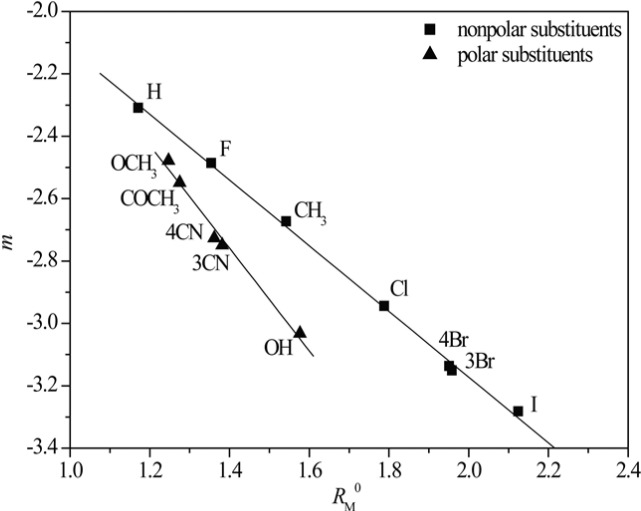
*R*_M_^0 ^- *m* dependency obtained in methanol

**Figure 2:**
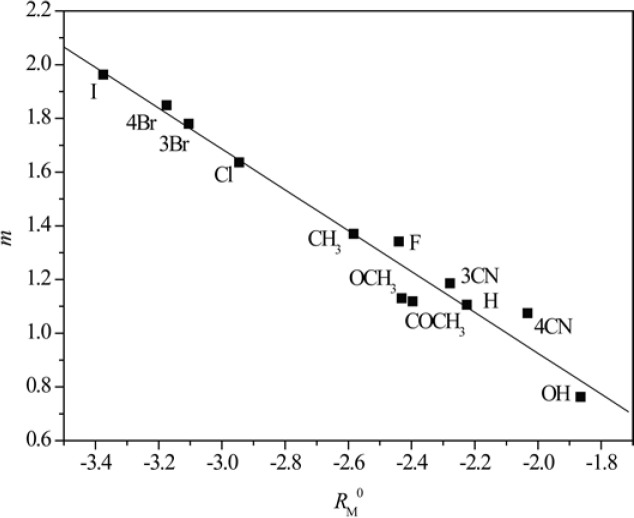
*R*_M_^0 ^- *m* dependency obtained in acetone

The presumption that the chromatographic parameters, *R*_M_^0^ and *m*, depend on the same physico-chemical characteristics is confirmed by high values of the regression coefficient of their linear correlation. This phenomenon was noted in literature, earlier (42). Additionally a separation of the compounds based on the polarity of substituents (Figure 1) was observed in methanol as modifier. This did not happen in acetone because it is less polar than the methanol, and thus less sensitive to detect the differences in the polarity of the substituents (Figure 2).

*Relationship between the standard measure of lipophilicity, *log* P, and chromatographic parameters, R*_M_^0^* and m*

With the idea to examine whether the chromatographic parameters, *R*_M_^0^, and *m* could be applied as valid measures of lipophilicity of the studied chloroacetamide derivatives, these two parameters were correlated firstly with the software calculated partition coefficient, log *P*, as a standard criteria of lipophilicity applying linear regression analysis. Derivatives with substituents at the position 3 were not included in correlations, because the mathematical procedure could not register the influence of substituent’s different position on the lipophilicity of compound. 

**Figure 3:**
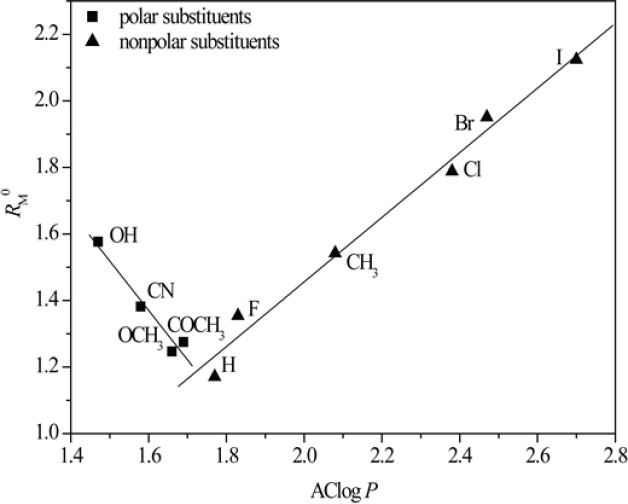
Relationship between *R*_M_^0^ values obtained in methanol and AClog *P.*

**Figure 4:**
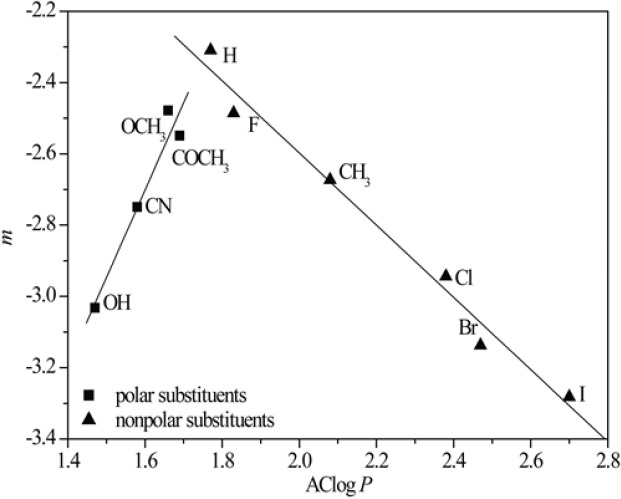
Relationship between *m* values obtained in methanol and AClog *P*

Figures 3 and 4 show obtained dependences AClog *P *- *R*_M_^0^ and AClog *P *- *m* in methanol as a modifier, respectively.

From Figures 3 and 4 the existence of linear dependence can be seen between experimentally (*R*_M_^0 ^and *m*) and mathematically (log *P*) determined lipophilicity of examined chloroacetamides. As previously noted, a grouping was again registered in methanol, of compounds based on the polarity of their substituent, which was not the case in acetone. 

In Table 6, the correlation matrix is given which was obtained as a result of the linear regression analysis between the chromatographic parameters of the examined compounds, *R*_M_^0 ^and *m*, determined in both modifiers used and various log *P*-values.

**Table 1:** Structures of the examined *N*-(substituted phenyl)-2-chloroacetamides

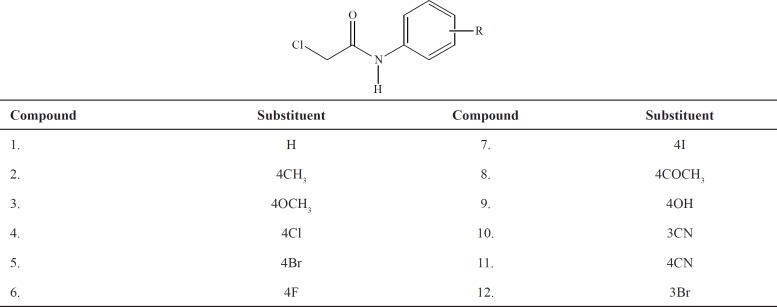

**Table 2:** Computed molecular descriptors of studied *N*-(substituted phenyl)-2-chloroacetamides

***R***	**MW**	**nON**	**nOHNH**	**natoms**	**MR**
H	169.611	2	1	19	43.76
4CH_3_	183.638	2	1	22	49.66
4OCH_3_	199.637	3	1	23	51.01
4Cl	204.056	2	1	19	48.37
4Br	248.507	2	1	19	51.46
4F	187.601	2	1	19	44.17
4I	295.507	2	1	19	56.26
4COCH_3_	211.648	3	1	24	55.01
4OH	185.61	3	2	20	45.58
3CN	194.621	3	1	20	49.86
4CN	194.621	3	1	20	49.86
3Br	248.507	2	1	19	51.46

MW: molecular weight; nON: number of hydrogen bond acceptor; nOHNH: number of hydrogen bond donor; natoms: the total number of atoms in molecule; MR: molar refractivity

**Table 3:** Software calculated log *P*-values of tested chloroacetamides

***R***	**AClog ** ***P***	**Alog ** ***P***	**Alog ** ***P***_s_	**Mlog ** ***P***	**milog ** ***P***	***kowwin***	**log ** ***P*** **ch.s**	**Xlog ** ***P***_3_
H	1.77	1.70	1.73	1.95	1.72	1.68	1.64	1.63
4CH_3_	2.08	2.18	1.87	2.25	2.17	2.23	2.31	1.99
4OCH_3_	1.66	1.68	1.69	1.68	1.78	1.76	1.86	1.67
4Cl	2.38	2.36	2.39	2.52	2.40	2.32	2.59	2.26
4Br	2.47	2.44	2.42	2.66	2.53	2.57	2.79	2.32
4F	1.83	1.90	2.00	2.37	1.88	1.88	1.91	1.73
4I	2.70	2.72	2.94	2.81	2.81	2.85	3.21	2.28
4COCH_3_	1.69	1.43	1.59	1.89	1.62	1.36	1.65	1.86
4OH	1.47	1.43	0.97	1.38	1.24	0.85	1.32	1.27
3CN	1.58	1.57	1.53	1.59	1.45	1.78	1.50	1.82
4CN	1.58	1.57	1.54	1.59	1.48	1.78	1.52	1.35
3Br	2.47	2.44	2.42	2.66	2.51	2.57	2.78	2.94

**Table 4:** Parameters of the chromatographic equations *R*_М_^0^*, m, *r and sd obtained for chloroacetamides in used modifiers.

***R***	**methanol**					**aceton** **e**		
	*R*_M_^0^	*m*	r	sd	*R*_M_^0^	*m*	r	sd
H	1.171 (±0.123)	-2.309 (±0.268)	0.980	0.046	1.106 (±0.132)	-2.225 (±0.285)	0.984	0.026
4CH_3_	1.542 (±0.067)	-2.673 (±0.146)	0.996	0.025	1.370 (±0.097)	-2.583 (±0.220)	0.989	0.028
4OCH_3_	1.247 (±0.122)	-2.478 (±0.265)	0.983	0.045	1.130 (±0.115)	-2.431 (±0.258)	0.983	0.033
4Cl	1.788 (±0.140)	-2.944 (±0.306)	0.984	0.052	1.636 (±0.139)	-2.945 (±0.313)	0.984	0.040
4Br	1.951 (±0.100)	-3.137 (±0.218)	0.993	0.037	1.849 (±0.087)	-3.175 (±0.195)	0.994	0.025
4F	1.354 (±0.088)	-2.486 (±0.192)	0.991	0.033	1.341 (±0.042)	-2.440 (±0.095)	0.998	0.012
4I	2.124 (±0.122)	-3.282 (±0.266)	0.990	0.046	1.963 (±0.143)	-3.375 (±0.323)	0.986	0.041
4COCH_3_	1.275 (±0.121)	-2.549 (±0.263)	0.984	0.045	1.118 (±0.077)	-2.396 (±0.173)	0.992	0.022
4OH	1.577 (±0.069)	-3.032 (±0.154)	0.997	0.025	0.763 (±0.030)	-1.865 (±0.068)	0.998	0.009
3CN	1.362 (±0.082)	-2.726 (±0.182)	0.996	0.030	1.186 (±0.101)	-2.278 (±0.218)	0.991	0.019
4CN	1.382 (±0.039)	-2.749 (±0.086)	0.999	0.014	1.074 (±0.117)	-2.033 (±0.263)	0.976	0.033
3Br	1.958 (±0.115)	-3.151 (±0.251)	0.991	0.043	1.780 (±0.105)	-3.105 (±0.238)	0.991	0.030

**Table 5:** Equations of *R*_M_^0 ^- *m* relationships of examined chloroacetamides in used modifiers

**modifier**	**equation**		**r**	**sd**
methanol	polar substituents	*R*_M_^0 ^= -0.450 – 1.649*m*	0.993	0.029
	nonpolar substituents	*R*_M_^0 ^= -1.065 – 1.053*m*	0.999	0.018
acetone	all substituents	*R*_M_^0 ^= -0.595 – 0.760*m*	0.981	0.075

**Table 6:** Correlation matrix between various log *P*-values and chromatographic parameters, *R*_M_^0 ^and *m*.

**log ** ***P***	**r**					
	**methanol**				**acetone**	
	**polar substituents**		**nonpolar substituents**		**all substituents**	
	***R***_M_^0^	***m***	***R***_M_^0^	***m***	***R***_M_^0^	***m***
AClog *P*	0.976	0.971	0.992	0.991	0.976	0.970
Alog *P*	---	---	0.990	0.985	0.958	0.934
Mlog *P*	0.876	0.860	0.946	0.944	0.957	0.929
milog *P*	0.973	0.985	0.996	0.992	0.982	0.981
Alog *P*s	0.969	0.950	0.937	0.934	0.977	0.957
log *P*ch.s	0.940	0.960	0.994	0.990	0.976	0.981
*kowwin*	0.764	0.732	0.987	0.983	0.954	0.910
Xlog *P*_3_	0.844	0.858	0.955	0.958	0.927	0.958

**Table 7:** Calculated pharmacokinetic predictors of the examined chloroacetamides

***R***	***P***_eff_	***PPB***	**log ** ***BBB***
H	3.536	45.46	0.20
4CH_3_	4.243	76.38	0.31
4OCH_3_	3.254	64.10	0.13
4Cl	4.857	80.00	0.42
4Br	5.213	84.80	0.47
4F	4.559	63.51	0.24
4I	5.579	91.06	0.58
4COCH_3_	3.679	61.70	0.14
4OH	1.956	41.59	0.04
3CN	2.366	62.42	0.03
4CN	3.102	62.42	0.03
3Br	4.546	84.80	0.47

**Table 8.** Correlation matrix between important pharmacokinetic predictors and chromatographic parameters, *R*_M_^0 ^and *m*

**pharmacokinetic predictor**	**r**					
	**methanol**				**acetone**	
	**polar substituents**		**nonpolar substituents**		**all substituents**	
	***R***_M_^0^	***m***	***R***_M_^0^	***m***	***R***_M_^0^	***m***
*P*_eff_	0.940	0.914	0.940	0.938	0.964	0.934
*PPB*	0.932	0.903	0.952	0.945	0.929	0.921
log *BBB*	0.796	0.832	0.992	0.990	0.966	0.967

**Figure 5:**
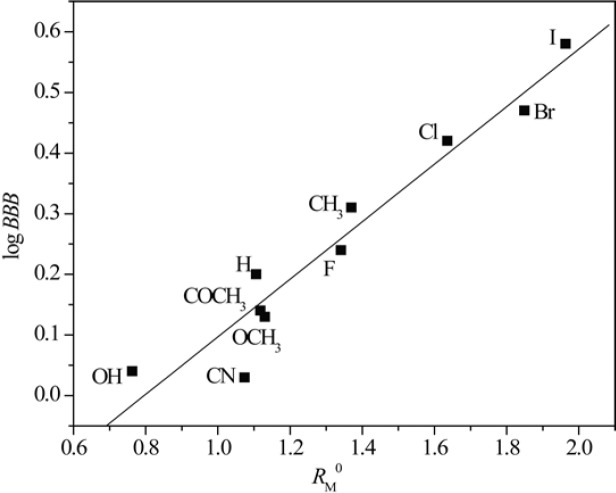
Relationship between *R*_M_^0^ values obtained in acetone and log *BBB*

**Figure 6:**
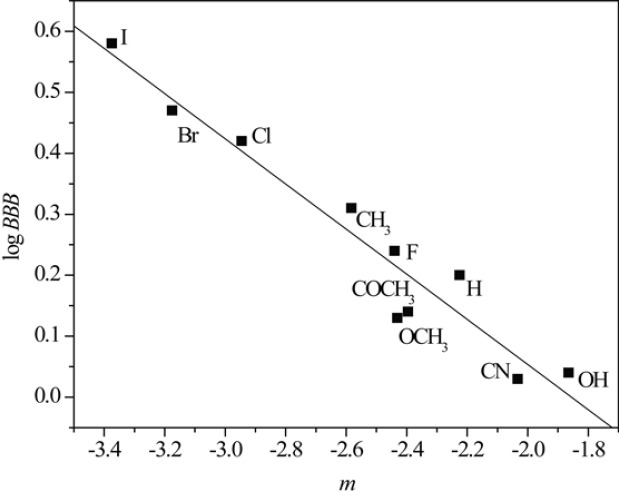
Relationship between *m* values obtained in acetone and log *BBB*

**Figure 7:**
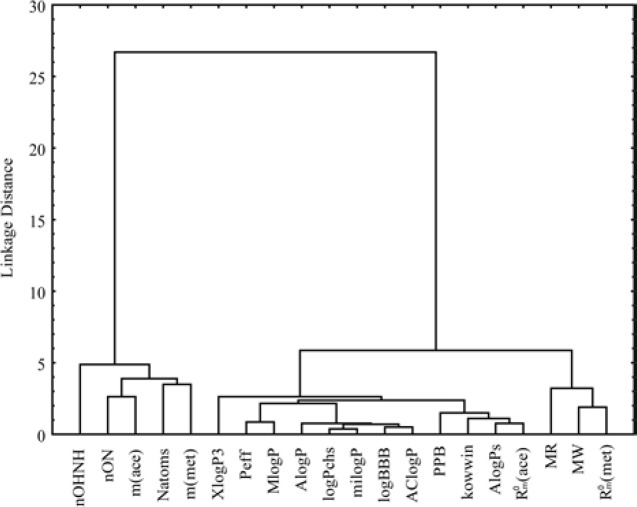
Dendrogram of bioactivity parameters of examined chloroacetamides

**Figure 8:**
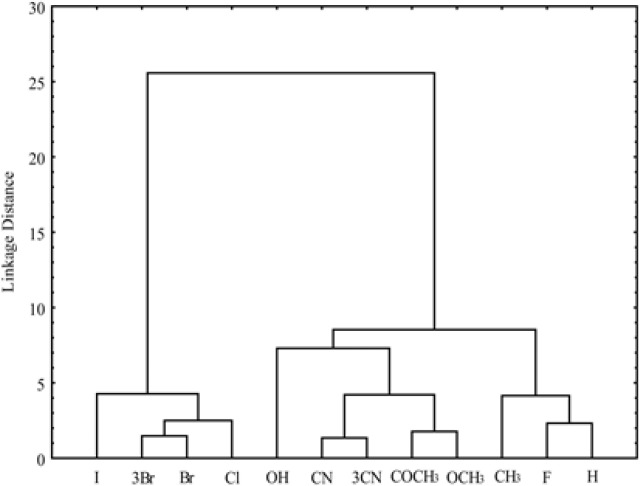
Dendrogram of examined derivatives chloroacetamides based on their parameters of bioactivity

**Figure 9:**
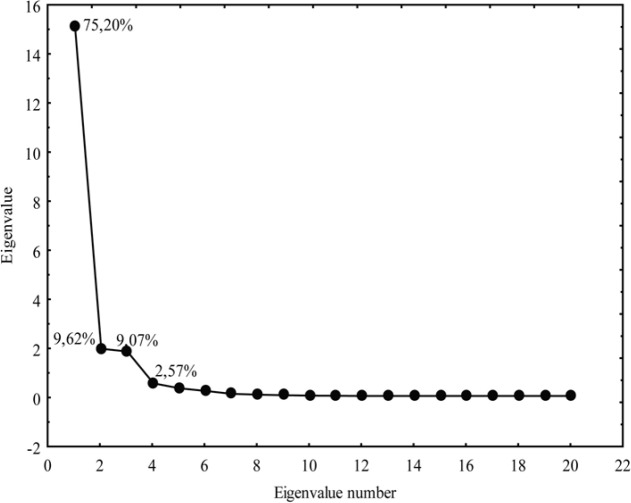
Eigenvalues of correlation matrix for the examined chloroacetamides based on the parameters of bioactivity

**Figure 10:**
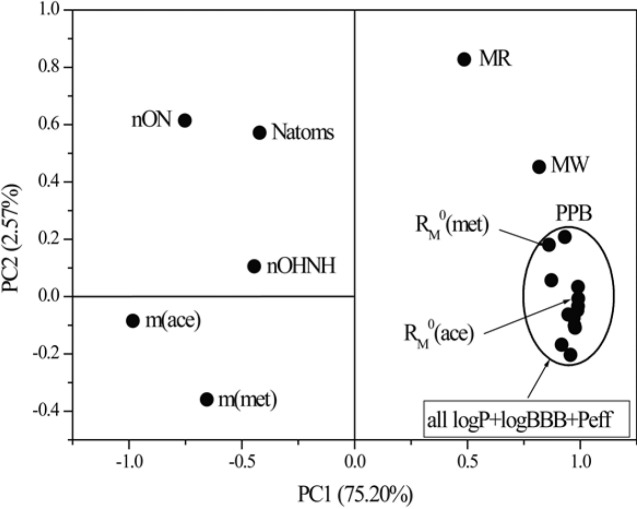
Loading plots as a result of PC1 versus PC2

**Figure 11:**
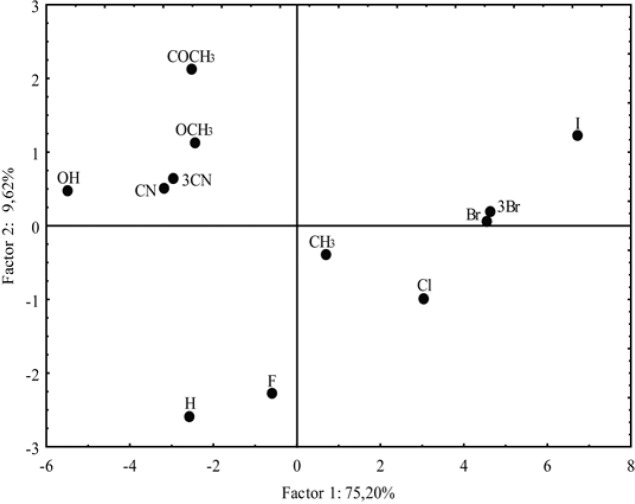
Score plots as a result of PC1 versus PC2

**Figure 12:**
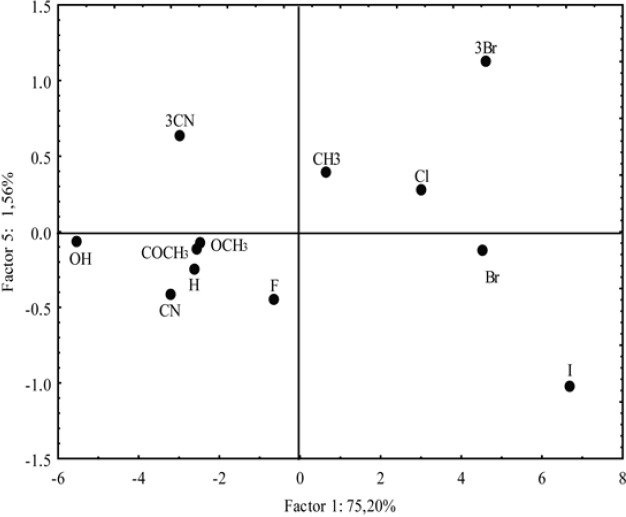
Score plots as a result of PC1 versus PC5

The best agreement with the experimentally determined lipophilicity, parameters *R*_M_^0 ^and *m* was shown by AClog *P* among all the calculated values of the partition coefficients and the lowest correlation on average was shown by *kowwin*.

*Determination of the pharmacokinetic predictors of chloracetamide derivatives*

The early stages of rational design of the biologically active agents require knowledge and understanding of their pharmacokinetics. ADME properties of the compound are crucial for assessing the effectiveness in biological medium (43). One of the most frequent reasons for failure of the bioactive candidates in clinical development is their poor bioavailability. Bioavailability represents the ratio of the unchanged drug administered by the route of interest and after administration direct into the systemic circulation (44). Therefore, it is expected that intravenous administration is preferred (100% bioavailability), but this is often due to practical reasons replaced by oral administration. Consequently, the biologically active compound is subjected to various changes before reaching the site of action and it is necessary to have information about its intestinal absorption. This represents the passage of the compound across the outer mucosal membranes of the gastrointestinal tract (45). The pharmacokinetic parameter that characterizes the rate of human intestinal absorption of compounds after oral administration is the human effective permeability in jejunum, *P*_eff_ (46). Compounds diffuse through the membrane of enterocytes so their permeability, and hence *P*_eff_ values increase with their lipophilicity (47).

After reaching the systemic blood circulation, the compound could be more or less bound to plasma proteins. The ratio of its concentration bound to the protein and its total concentration is defined as protein plasma binding affinity, *PPB*. 

Only free fraction of the compound passes through the cell membrane and binds to the appropriate molecular target (48). Hence, the compound’s activity and efficiency depend on the degree and the extent of binding to the plasma proteins. The bound fraction correspond depot which release compound slowly as the unbound form when the unbound form is metabolized. The compound that acts in the central nervous system (CNS) needs to cross the blood–brain barrier (BBB). The possibility of the application of the compound as a potential neurological agent is delineate by the ratio of drug concentration in the brain and drug concentration in the blood log *BBB* (49). Compounds with log* BBB* > 0.3 pass through blood brain barrier readily, while log *BBB* < -1 indicates a blocking of the penetration (50). 

Taking into account the significance of knowing the pharmacokinetics in predicting bioactivity of substances, some important pharmacokinetic predictors of the tested chloroacetamide derivatives were calculated applying the software packages Chemsilico and Simulation (Table 7). 

As can be seen from Table 7, the most lipophilic derivative (-I as substituent) has the highest value of *P*_eff _and as it was expected the lowest absorption evince the most polar compound (-OH group as substituent). Furthermore, while reviewing the values of *PPB,* it was noticed that the compound with -I as substituent exhibits the greatest affinity for binding to plasma proteins. The largest amounts of the unbound compound arrive to the surrounding tissue in the case of derivative with -OH group. Also, the derivatives with alkyl and halogen substituents show the greatest possibility of application as neurological agents (log* BBB* > 0.3).

*Correlation of pharmacokinetic predictors and chromatographic parameters, R*_M_^0^ *and m*

Taking into account the fact that the lipophilicity of the compound is closely related to its pharmacokinetics, chromatographic parameters, *R*_M_^0^ and *m*, as the presumed measures of lipophilicity of the tested chloroacetamides were correlated with three pharmacokinetic predictors *P*_eff_, *PPB* and log *BBB *applying linear regression analysis. Figures 5 and 6 illustrate the obtained correlations of *R*_M_^0^ and *m* determined in acetone with pharmacokinetic predictor log *BBB* and results of all established relationships are presented in Table 8.

Figures 5 and 6 as well as data in Table 8 confirm that linear regression analysis gave satisfactory correlations of *R*_M_^0^ and *m* with the selected pharmacokinetic predictors. Also, again it can be observed separating of compounds in methanol which is previously explained. 

From results represented in Table 8, it can be seen that the chromatographic parameter, *R*_M_^0^ obtained in both used modifiers achieve better relationships with all pharmacokinetic predictors than parameter *m*. Also, among all the calculated predictors, *P*_eff _gave the highest agreement with both chromatographic parameters_. _The obtained results indicates that RPTLC parameters could be used successfully in preliminary tests of potential bioactivity of *N*-(substituted phenyl)-2-chloroacetamides.

*Establishing relationships between different indicators of bioactivity applying multivariate methods*

In addition to the method of linear regression, in order to gain a better insight into the relationship between the investigated molecular descriptors, experimentally determined measures of lipophilicity, chromatographic retention parameters, *R*_M_^0^and *m*, were interconnected with the standard measure of lipophilicity, the partition coefficient, log *P*, important pharmacokinetic predictors and parameters of Lipinski and Ghose, respectively, by applying multivariate analysis. Multivariate methods enable the analysis and classification of numerous experimental results obtained in different ways and the establishing of the relationship between them based on their (dis)similarities. Among the methods of multivariate analysis significantly stand out cluster analysis, CA, and principal component analysis, PCA (51-55). 

The data matrix for performing CA and the PCA analysis consists of the columns (variables) which correspond to experimentally and mathematically obtained lipophilicity, pharmacokinetic predictors, parameters of Lipinski and Ghose, while examined chloroacetamide derivatives represented the rows (cases). Prior to the analysis, the data matrix was standardized, because the analyzed data were measured on different scales.

As the measure of dissimilarity between objects, in CA the Euclidean distance was used, while the Ward’s linkage method was applied for testing the linkage measure. As results of cluster analysis for different parameters of bioactivity was obtained dendrogram which is shown in Figure 7 and the other one for the investigated chloroacetamides presented in Figure 8.

Figure 7 shows the separation of the analyzed data into two clusters. The first cluster contains the chromatographic parameter *m *(obtained in both used modifiers) and parameters of Lipinski and Ghose which mainly refer to the number of atoms (total, hydrogen bond acceptors, hydrogen bond donors). This confirmed the already known phenomenon that the parameter *m* is directly related to the characteristics of solute (39).

In the second cluster the rest of lipophilicity parameters are placed, all pharmacokinetic predictors and MW, MR. Inside the second cluster subcluster can be noticed, which contain MW, MR and chromatographic constant *R*_M_^0^ obtained in methanol. Separation of MW and MR from other parameters of Lipinski and Ghose confirms the fact that these two properties are more related to biomolecular interactions, hence theoretically influences the lipophilicity (MW) and pharmacokinetic behavior (MR) of the compound (56). Also, it is noticeable that the apartness of the chromatographic parameter *m *from chromatographic retention constant *R*_M_^0^ and log *P* what was registered in the literature earlier (57). This grouping of the obtained lipophilic parameters indicates a higher similarity of the chromatographic retention constant *R*_M_^0 ^to the log *P* and a greater influence on the pharmacokinetic predictors than the chromatographic parameter *m* at given conditions. The cluster analysis of chloroacetamides which can show the influence of nature of the substituents in the molecules on their bioactivity, was resulted with dendrogam which has two clearly defined clusters (Figure 8). In the first cluster derivatives having halogen as substituent are grouped, while the second contains the rest of derivatives. From Figure 8 it can also be noted that the second cluster is divided into two subclusters. One subclusters includes the compounds with polar substituents (-OH, -CN, 3CN, COCH_3_, OCH_3_) and in the other are the unsubstituted compound and derivatives with -CH_3_ and -F as substituents. The derivative with -F as substituent is secluded from other derivatives with halogen substituents given which has a high polarization force.

Besides the cluster analysis, the principal component analysis was conducted. It is a significant multivariate method since it provides identification and elimination of the redundant data from the experimental results, therefore ensuring the required reduction in their number with a minimal wastage of information. By PCA the original data matrix was decomposed into loading (lipophilic parameters, pharmacokinetic predictors, parameters of Lipinski and Ghose) and score (tested chloroacetamides) vectors, whereby new variables-principal components were obtained (Figure 9).

As can be observed from Figure 9, five principal components have described 98.02% (PC1 75.20%, PC2 9.62%, PC3 9.07%, PC4 2.57% and PC5 1.56%) of total variance in the data. 

By comparing the obtained PCs of variables (parameters of Lipinski and Ghose, experimentally and mathematically determined lipophilicity and pharmacokinetic predictors), the loading plots were obtained (Figure 10). 

This dependence of the two first principal components resulted in the separation of the examined descriptors into two characteristic groups based on the PC1 values. One group is described by the negative PC1 and includes *m* values, nON, nOHNH and natoms thus demonstrating already observed connection between them. The second involves mathematically obtained log *P-*values, chromatographic parameters *R*_M_^0^ determined in both used modifiers, pharmacokinetic predictors, MW and MR (positive PC1). By this has been confirmed again that chromatographic constant *R*_M_^0^ corresponds to the parameter log *P* and to the selected pharmacokinetic predictors in greater extent than parameter *m*. Such distribution of parameters confirmed the results previously detected by the cluster analysis. As can be seen from Figure 10 within the existing group, the PC2 value can classify the parameters in finer way according to the nature of the information they carry. The chromatographic parameter *m* obtained in both applied modifiers has negative PC2 and stand apart from the theoretical parameters relating to the number of atoms. On the other hand a very close grouping can be observed of chromatographic constant, *R*_M_^0^ obtained in both of the used modifiers with standard lipophilic parameters and pharmacokinetic predictors. A slightly greater similarity is thereby registered in the case of *R*_M_^0^ obtained in acetone than in methanol. Descriptors MW and MR in the loading plots appear as outliers.

In addition, the PCA enabled important classification of the investigated chloroacetamide derivatives based on differences in their lipophilicity and pharmacokinetics. The correlation of the two main components for the analyzed chloroacetamides (score plot) is illustrated in Figure 11. The nature of the substituent -R caused grouping of the compounds which was registered in literature (58).

Differences between them are reflected in the value of PC1. One group includes derivatives with polar substituents and negative values of PC1, while the second group encompasses compounds with non-polar substituents (except -H and -F) and they are characterized by the positive PC1. The most negative value of PC1 has a compound with -OH group as a substituent and the most positive PC1 is registered for the most lipophilic compound, with more voluminous halogen substituent (-I as substituent). It is obvious that the compounds with the negative value PC1 at the same time are divided based on the value of PC2. Thus, derivatives with polar substituents (positive PC2) are separated from the compounds with -H and -F (negative PC2). Again, the results obtained by the PCA analysis are very similar to the CA results, but the PCA is more subtle given that the derivative with a -CH_3_ substituent is classified as lipophilic. 

Other interdependencies between the main components did not give any significant distribution of the examined compounds. However, regardless of the small value of the PC5 (1.56%) the important distribution of the analyzed chloroacetamides by PC1-PC5 correlation was registered (Figure 12).

Namely, derivatives with -Br and -CN in position 3 (positive PC5) are separated from the derivatives with same substituent in position 4 (negative PC5). Thereby, it is confirmed that the additional advantage of the PCA is representing its high sensitivity for detecting extremely small differences between the compounds with the same substituent in different positions in which the experimental and theoretical methods for determining lipophilicity with sufficient visibility could not be done. 

## Conclusion

Derivatives of *N*-(substituted phenyl)-2-chloroacetamides were studied with the goal of predicting their potential biological activity by fulfilling the rules of bioactivity (Lipinski and Ghose) and determining their lipophilicity, experimentally by RPTLC in presence of methanol and acetone, and computationally by applying software package. 

By applying the classic linear regression analysis and chemometric methods (CA and PCA) the presumed measures of lipophilicity (biological activity) such as chromatographic retention parameter, *R*_M_^0 ^and *m*, were found to be in a good relationship with partition coefficient, log *P*, as a standard criteria of lipophilicity (average correlation coefficient, r = 0.947) and with significant pharmacokinetic predictors, *P*_eff_, *PPB* and log *BBB* (average correlation coefficient, r = 0.931). More concretely, the chromatographic retention constant, *R*_M_^0 ^gave stronger dependencies under the existing conditions with the used descriptors than parameter *m*. In addition, the multivariate methods provided an essential sensitivity for the distribution of data according to their similarities and differences which could be decisive in the preliminary tests of the biological potential of the compounds. 

The results of all the conducted correlation methods pointed that the chromatographic parameters, *R*_M_^0 ^and *m*, determined by applying RPTLC offer the possibility for a more complete understanding and reliable prediction of properties (lipohilicity, pharmacokinetics, bioavailability) which indicate the biological activity of *N*-(substituted phenyl)-2-chloroacetamides at the earliest phase of modern design of bio agents. 
